# Horizontal Gene Transfer and CRISPR Targeting Drive Phage-Bacterial Host Interactions and Coevolution in “Pink Berry” Marine Microbial Aggregates

**DOI:** 10.1128/aem.00177-23

**Published:** 2023-07-05

**Authors:** James C. Kosmopoulos, Danielle E. Campbell, Rachel J. Whitaker, Elizabeth G. Wilbanks

**Affiliations:** a Department of Bacteriology, University of Wisconsin-Madison, Madison, Wisconsin, USA; b Microbiology Doctoral Training Program, University of Wisconsin-Madison, Madison, Wisconsin, USA; c Microbial Diversity 2020, University of Chicago Marine Biological Laboratory, Falmouth, Massachusetts, USA; d Department of Medicine, Division of Infectious Diseases, Washington University School of Medicine, St. Louis, Missouri, USA; e Edison Family Center for Genome Sciences & Systems Biology, Washington University School of Medicine, St. Louis, Missouri, USA; f Department of Ecology, Evolution, and Marine Biology, University of California, Santa Barbara, California, USA; g Department of Microbiology, University of Illinois, Urbana, Illinois, USA; h Carl R. Woese Institute for Genomic Biology, University of Illinois, Urbana, Illinois, USA; Unversidad de los Andes

**Keywords:** CRISPR, horizontal gene transfer, metagenomics, microbial ecology, microbial evolution, phage, viromics

## Abstract

Bacteriophages (phages), which are viruses that infect bacteria, are the most abundant components of microbial communities and play roles in community dynamics and host evolution. However, the study of phage-host interactions is hindered by a paucity of model systems from natural environments. Here, we investigate phage-host interactions in the “pink berry” consortia, which are naturally occurring, low-diversity, macroscopic bacterial aggregates that are found in the Sippewissett Salt Marsh (Falmouth, MA, USA). We leverage metagenomic sequence data and a comparative genomics approach to identify eight compete phage genomes, infer their bacterial hosts from host-encoded clustered regularly interspaced short palindromic repeats (CRISPRs), and observe the potential evolutionary consequences of these interactions. Seven of the eight phages identified infect known pink berry symbionts, namely, *Desulfofustis* sp. PB-SRB1, *Thiohalocapsa* sp. PB-PSB1, and *Rhodobacteraceae* sp. A2, and they are largely divergent from known viruses. In contrast to the conserved bacterial community structure of pink berries, the distribution of these phages across aggregates is highly variable. Two phages persisted over a period of seven years with high sequence conservation, allowing us to identify gene gain and loss. Increased nucleotide variation in a conserved phage capsid gene that is commonly targeted by host CRISPR systems suggests that CRISPRs may drive phage evolution in pink berries. Finally, we identified a predicted phage lysin gene that was horizontally transferred to its bacterial host, potentially via a transposon intermediary. Taken together, our results demonstrate that pink berry consortia contain diverse and variable phages as well as provide evidence for phage-host coevolution via multiple mechanisms in a natural microbial system.

**IMPORTANCE** Phages, which are viruses that infect bacteria, are important components of all microbial systems, in which they drive the turnover of organic matter by lysing host cells, facilitate horizontal gene transfer (HGT), and coevolve with their bacterial hosts. Bacteria resist phage infection, which is often costly or lethal, through a diversity of mechanisms. One of these mechanisms is CRISPR systems, which encode arrays of phage-derived sequences from past infections to block subsequent infection with related phages. Here, we investigate the bacteria and phage populations from a simple marine microbial community, known as “pink berries”, found in salt marshes of Falmouth, Massachusetts, as a model of phage-host coevolution. We identify eight novel phages and characterize a case of putative CRISPR-driven phage evolution as well as an instance of HGT between a phage and its host, together suggesting that phages have large evolutionary impacts in a naturally occurring microbial community.

## INTRODUCTION

Phages, viruses that infect bacteria, occur in all microbial ecosystems, often outnumbering bacteria by 10 to 1, and play pivotal roles in altering community structure (1–3), mediating horizontal gene transfer (HGT) (3–5), and driving bacterial evolution (6–8). Although some phage-host interactions can be beneficial, phage infection canonically ends with the lysis and death of the host to release progeny phage particles for transmission to new host cells. Thus, there is strong selection for bacteria to evolve mechanisms by which to resist infection. Likewise, phages must evolve to overcome those resistances to survive. This coevolution between phages and bacterial hosts is often described as an “arms race” (9).

Bacteria have evolved a wide range of phage defense systems, such as clustered regularly interspaced short palindromic repeat (CRISPR) loci, which act as microbial adaptive immune systems. During a new phage infection, CRISPR systems incorporate short segments of a phage-derived sequence, known as “protospacers,” into CRISPR arrays as “spacers” (10–12). CRISPR systems further encode mechanisms to degrade invading phage DNA that matches an existing spacer, thereby allowing the host to resist infection. Thus, CRISPR arrays serve as a genetic record of the phages that a host has encountered and can be leveraged to identify phage hosts from sequence data (13, 14).

An in-depth analysis of CRISPR targeting offers insights into phage-host interactions. CRISPR systems often target conserved phage sequences, thereby conferring protection from groups of related phages (10, 15, 16). Phages can accumulate mutations within protospacers, and this allows the phage to escape CRISPR defenses (1, 15, 17). Together, this variation in CRISPR spacer and protospacer nucleotide sequences can shed light on phage-host coevolution.

Here, we investigate phage-bacterial interactions in microbial consortia known as “pink berries”. Pink berries are macroscopic microbial aggregates that are found in the Sippewissett Salt Marsh of Falmouth, MA (18, 19). These aggregates are primarily composed of two species of phototrophic, sulfide-oxidizing, and sulfate-reducing bacteria that together form a syntrophic sulfur cycle (19). Although phage sequences have previously been found in pink berries (20), the interactions between phages and their hosts remain uncharacterized. We show that pink berries host diverse, novel phages that drive bacterial evolution through HGT and putative CRISPR-driven arms race dynamics.

## RESULTS

### Pink berries contain novel phages that are variable between individual aggregates.

**FIG 1:**
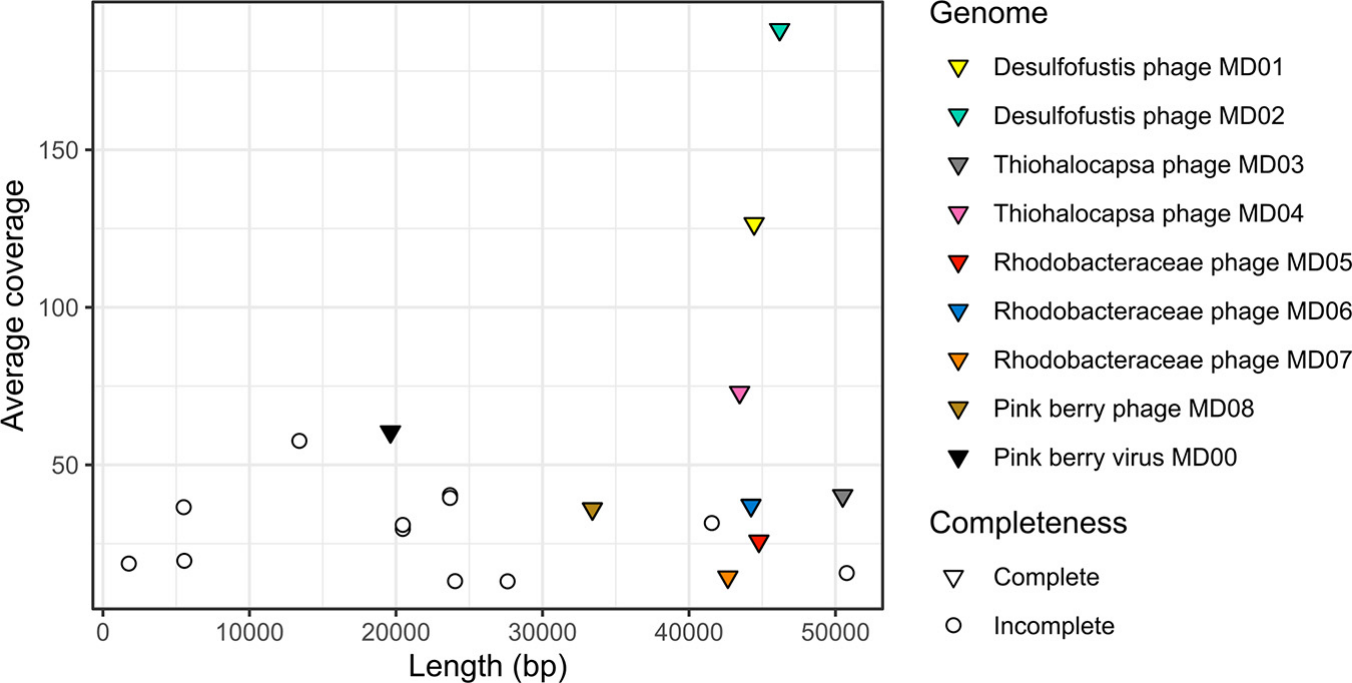
Coverage and length of coassembled pink berry viral contigs. Viral contigs were identified by using VIBRANT (21) and ViralVerify (22), and their genome completeness was assessed by using ViralComplete (22). Coverage values were calculated using the merged set of filtered and trimmed reads used for metagenome coassembly.

Pink berries were strategically sampled and pooled during sequencing library preparation to observe both the breadth of diversity across individual pink berries and to deeply sample the total diversity of the pink berries. Thus, we sequenced two metagenomes from single pink berries, one with a low sequencing depth (LS06-2018-s01) and one with a high sequencing depth (LS06-2018-s02), as well as one metagenome from three pink berries that were homogenized together and sequenced at a very high depth (LS06-2018-s03) (Table 1). The coassembly of the three pink berry metagenomes yielded 184 contigs, totaling 4.35 Mb in length (Table 1). Two phage sequence prediction tools, namely, Virus Identification By iteRative ANnoTation (VIBRANT) (21) and ViralVerify (22), identified nine full-length, circular phage genomes, and these were the targets of our downstream analyses (Fig. 1; Data Set S1). The phages were named according to their hosts predicted by CRISPR spacer-protospacer matches (described in the “Pink berry phages are targeted by bacterial CRISPR systems” subsection).

**TABLE 1:** Summary of pink berry metagenome sequencing and coassembly

Property	LS06-2018-s01	LS06-2018-s02	LS06-2018-s03
Total read length (Gb)	0.41	1.07	7.19
Quality trimmed total read length (Gb)	0.35	0.91	6.94
Percentage of total read length after quality trimming	87%	85%	97%
	Coassembly		
Contigs	184		
*N*_50_ (bp)	50,490		
L50	23		
Average read coverage (±SD)	12.89 ± 39.63		

**FIG 2:**
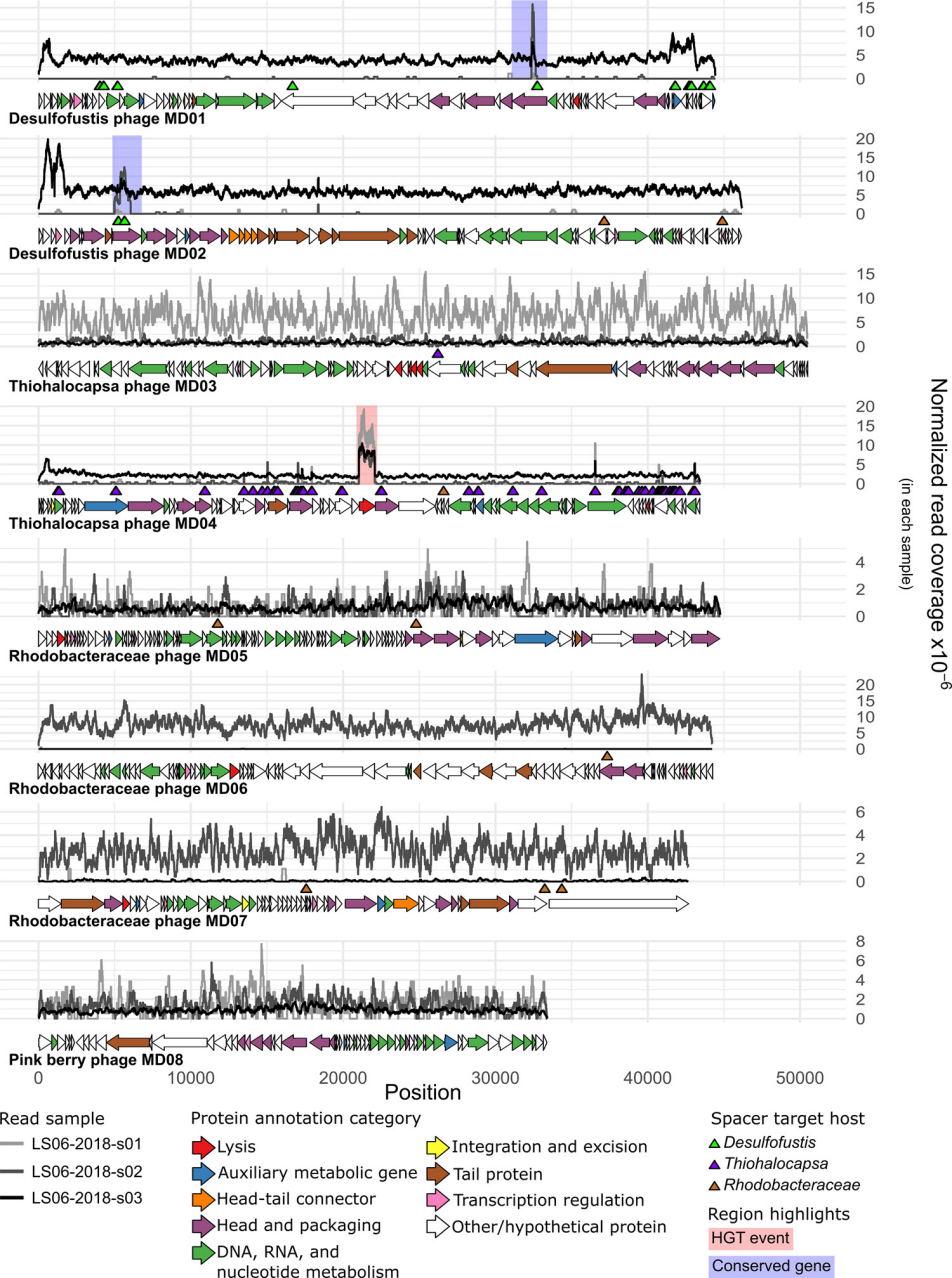
Complete phage genomes vary in abundance across samples and are targeted by bacterial CRISPR spacers. Normalized read coverage by position for each sample are given. Coverage values were normalized to the total number of trimmed and filtered reads for each sample. The horizontal arrows indicate the ORFs predicted by PHANOTATE (54), and their colors correspond to their predicted functional categories. The triangles indicate the genome positions of protospacers, and they are colored according to the host taxonomy of the corresponding spacer. Regions highlighted with a blue background indicate a conserved gene between phage genomes, as inferred by Clinker (62). Regions highlighted with a red background were found to be an HGT event between the phage and the host.

Functional annotations were obtained for 236 of 632 putative protein-coding genes that were predicted across the nine complete viral genomes (Data Set S2). One virus was found to have large amounts of homology to eukaryote-infecting circular Rep-encoding single-stranded (CRESS) DNA viruses and was excluded from further analysis to focus on the primary pink berry bacterial components and their phages (see the “Pink berry virus MD00” subsection) (Data Sets S1 and S2). Functional annotations for the remaining eight phage genomes predicted nucleotide metabolism proteins, head and packaging proteins, integration and excision proteins, transcriptional regulators, and various lytic proteins (Fig. 2; Data Set S2). All of the phages of interest here were predicted to exhibit strictly lytic lifecycles by BACPHLIP (23), and their lack of genes is required for a temperate life cycle (e.g., integrases).

There were also predictions for several auxiliary viral genes (24), which we inferred based on functional predictions outside the core functions required for the phage life cycle. We identified numerous orphan DNA methyltransferases (MTases) in the genomes of these phages (Data Set S3A), including homologs of C-5 cytosine MTases and N-4 cytosine/N-6 adenine MTases. Pink berry bacteria have numerous active restriction-modification systems (20) with predicted restriction sites in these phage genomes (Data Set S3B). Although the recognition sequences of these divergent phage MTases could not be predicted, phage-encoded MTases often confer resistance to host restriction enzymes by disguising recognition sites in the phage genome (25, 26). The presence of auxiliary genes, such as MTases, suggests that pink berry phages have adapted to avoid their hosts’ defenses, and this is consistent with an arms race-like process of coevolution.

**FIG 3:**
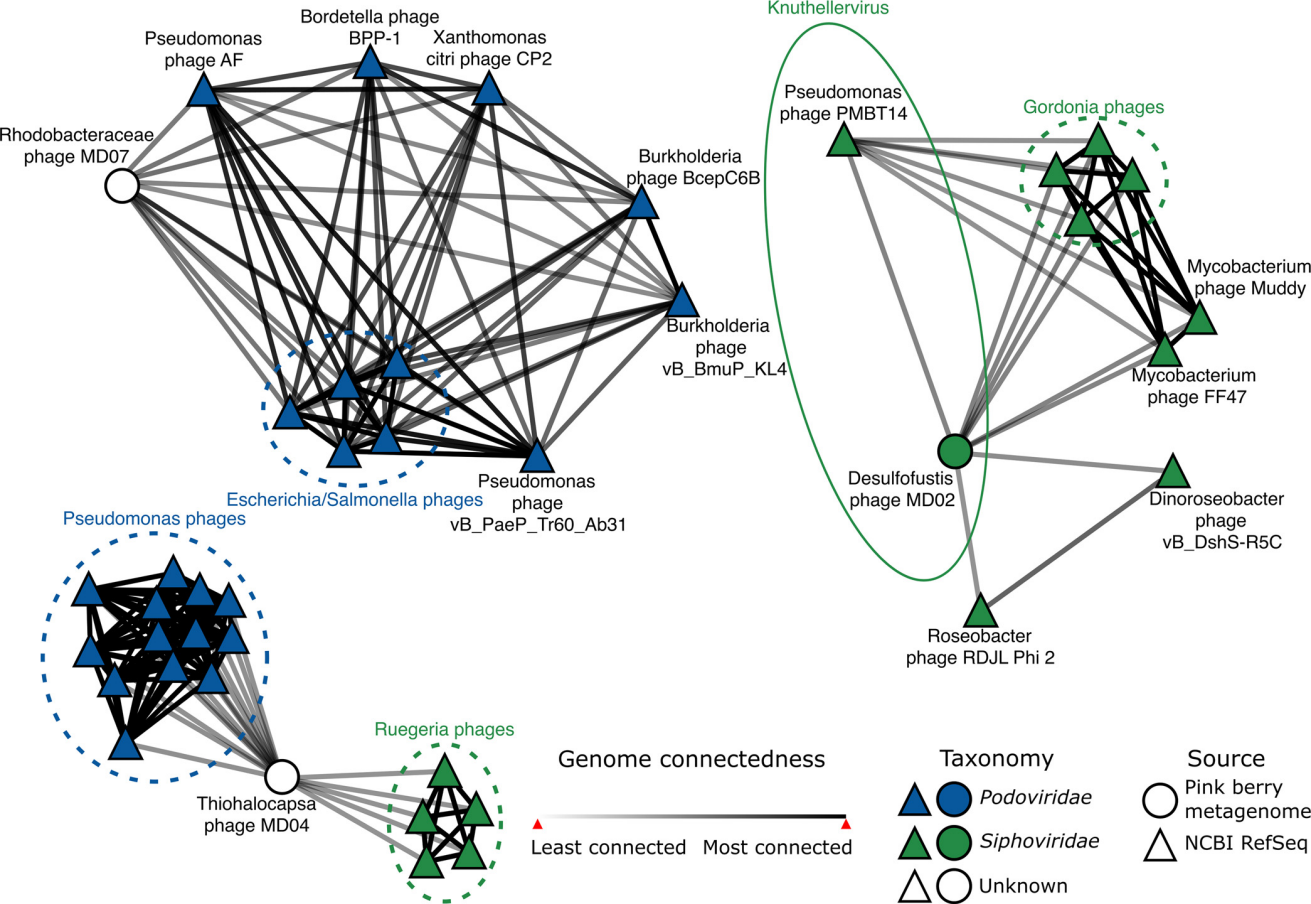
Pink berry consortia host largely novel phages. Each node represents a phage genome, and the edges represent genome relatedness, as inferred by vConTACT (28). The reference genomes are from the RefSeq Prokaryotic virus database v211 (52). The group circled with a solid line shows the phage genus of interest, namely *Knuthellervirus*. Groups circled in dashed lines show related phages infecting the same host. The network was visualized in Cytoscape v3.9.0 (78). Only phages which are first neighbors to pink berry phage genomes are shown.

Phage taxonomy is based on genome similarity (27) and forms the basis upon which to inferr the diversity of phages within a community. We hypothesized that pink berry phages would be at least as diverse as their pool of potential bacterial hosts, which span multiple phyla. vConTACT (28), which is a genome-wide protein similarity-based approach, was used to infer the phage taxonomy for the eight phage genomes of interest. Although nominal protein similarity was detected between all phages of interest and the Viral RefSeq protein database during gene annotation (Data Set S2), only Desulfofustis phage MD02, Thiohalocapsa phage MD04, and Rhodobacteraceae phage MD07 were connected to a known phage in the vConTACT network (Fig. 3; Data Set S4). Further, only one phage genome, namely, Desulfofustis phage MD02, had sufficient protein similarity to cluster with a cultured phage reference genome, namely, Pseudomonas phage PMBT14, which is currently the only species of the genus *Knuthellervirus* (Data Set S4). None of the phage genomes of interest clustered with each other. The remaining five genomes of interest lacked sufficient protein similarity for a connection in the vConTACT network, indicating that these phages represent novel and undescribed diversity.

**FIG 4:**
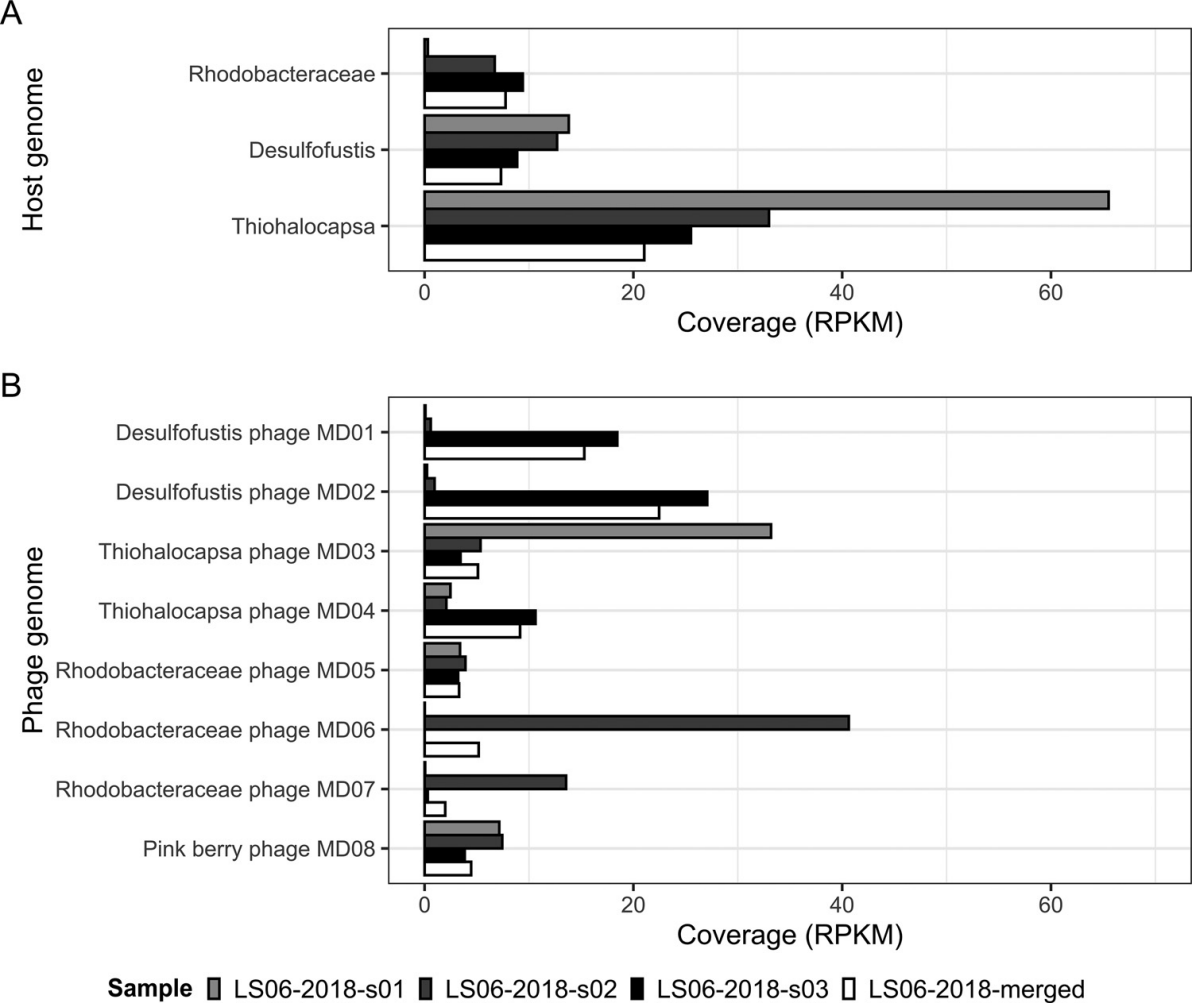
Phage presence and abundance are highly variable between pink berry communities. Average read coverages for host (A) and phage (B) genomes were converted to reads per kilobase million (RPKM) by using the total number of filtered and trimmed reads per sample.

Pink berry aggregates form a stable consortium with consistent community structure among their most abundant members (18–20). To assess the distribution of pink berry-associated bacteria and phages, genome-wide read coverages were analyzed for individual pink berry metagenomes (Fig. 4). In agreement with previous observations, we found that the relative abundance of pink berry bacteria is relatively homogenous across samples, with the exception of *Rhodobacteraceae* in one sample (Fig. 4A). In contrast, phage presence and abundance are highly variable between different pink berry samples and were not correlated with the abundance of their predicted hosts (Fig. 4B). Only two phages, namely, Rhodobacteraceae phage MD05 and pink berry phage MD08, are similarly abundant in each pink berry metagenome, whereas read mapping to the remaining six phages suggests that they are distributed unevenly between individual aggregates (Fig. 4).

Additionally, to observe whether the phage genomes of interest were present in pink berry metagenomes from previous years, we mapped reads from a metagenome of 10 pink berries sampled in 2011 (19). This revealed the nearly complete coverage of Desulfofustis phage MD01 and Rhodobacteraceae phage MD06 (Fig. S1), suggesting that these phages have persisted in pink berries for over seven years. The alignment of phage MD01 and MD06 to their related contigs from the assembly of the 2011 metagenome revealed that they share more than 97% and 99% average nucleotide identity (ANI), respectively. However, the visualization of these alignments, suggests that several gene gain and loss events have occurred in these phages over the seven years, including a predicted tail spike protein in Rhodobacteraceae phage MD06 that is absent from its presumed 2011 ancestor (Fig. S2). In contrast, read mapping of the 2011 data to Desulfofustis phage MD02 and Thiohalocapsa phage MD04 genome largely occurs at highly conserved regions and is likely the consequence of nonspecific cross-mapping (Fig. S1). Likewise, Thiohalocapsa phage MD03, Rhodobacteraceae phages MD05 and MD07, and pink berry phage MD00 had <1% or no genome coverage. Since neither this study nor the study from 2011 (19) are exhaustive surveys of pink berry diversity, it is difficult to determine the mechanisms behind the emergence of these six phages. Taken together, these results suggest that although pink berries have relatively simple and conserved bacterial community structures, their phages are highly variable over both space and time.

### Pink berry phages are targeted by bacterial CRISPR systems.

Bacterial CRISPR arrays serve as a record of past phage infections and can be used to infer hosts for phage genomes (13, 14). Two independent CRISPR spacer prediction tools identified a total of 48 unique repeat sequences from four reference genomes for known pink berry-associated bacteria: *Desulfofustis* sp. PB-SRB1 (GenBank: JAEQMT010000010.1), *Flavobacteriales* bacterium (GenBank: DNTB01000031.1), *Rhodobacteraceae* sp. A2 (GenBank: JAERIM010000001.1), and *Thiohalocapsa* sp. PB-PSB1 (GenBank: CP050890.1) (19). Parsing the remaining available pink berry genome, Oceanicaulis alexandrii sp. A1 (GenBank: JAERIO010000015.1) did not yield any CRISPRs. Because CRISPR repeat sequences are conserved within bacterial species (29, 30), they can be used to identify adjacent spacer sequences in unassembled metagenomic short reads (14, 31). Using the CRISPR repeat sequences from reference genomes, NARBL (14) identified 2,802 unique CRISPR spacer sequences from the set of merged metagenomic reads from LS06-2018-s01, LS06-2018-s02, and LS06-2018-s03. Of these, 798 spacers were adjacent to repeats that were associated with *Desulfofustis* sp. PB-SRB1. 71 were adjacent to *Flavobacteriales* repeats, 349 were adjacent to *Rhodobacteraceae* sp. A2 repeats, and 1,584 unique spacers were adjacent to repeats associated with *Thiohalocapsa* sp. PB-PSB1.

**FIG 5:**
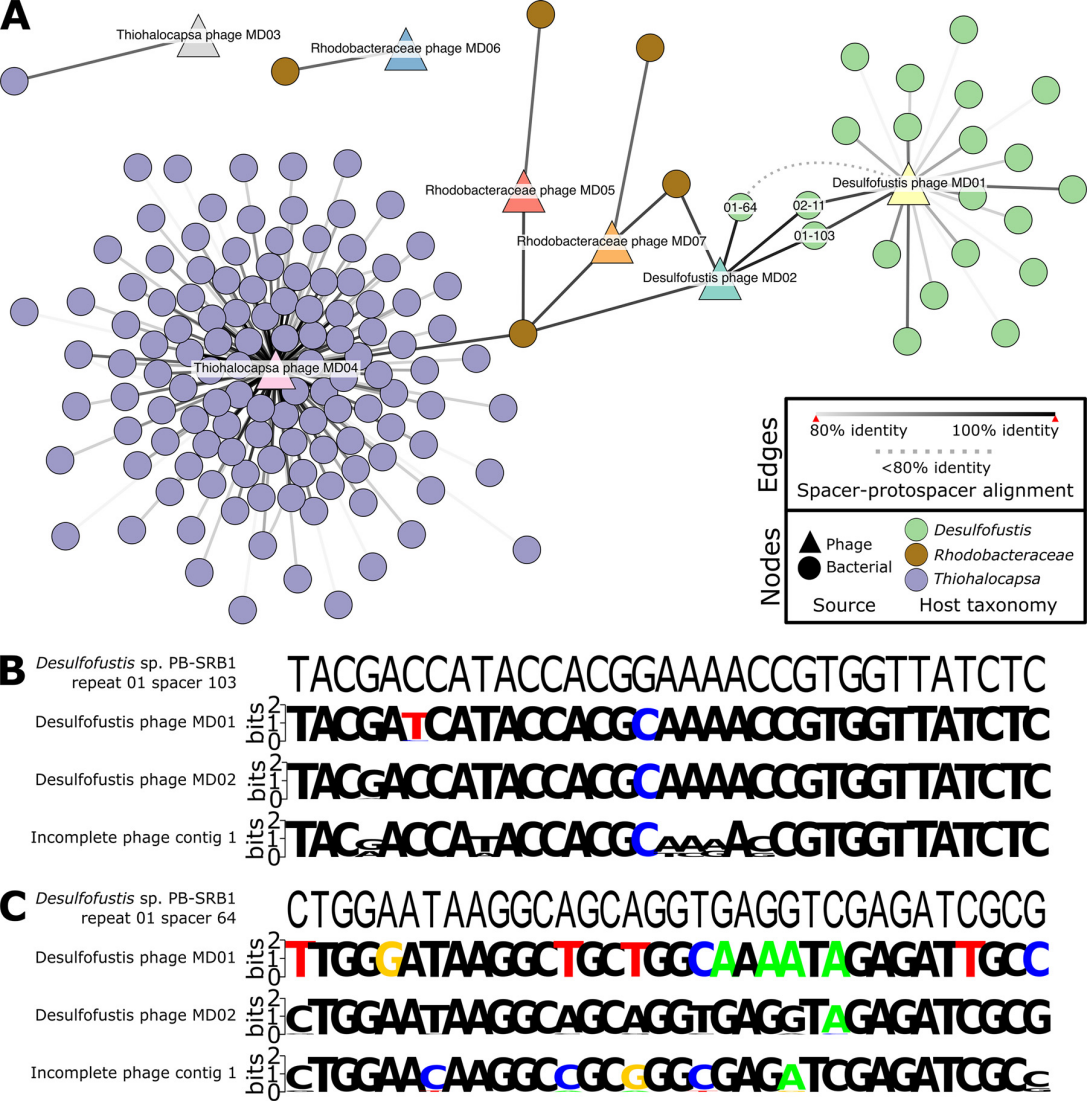
CRISPR spacer-phage genome alignments reveal hosts and a conserved protospacer. (A) Spacerblast (71) alignment results with at least 80% nucleotide identity over the entire spacer length were visualized in Cytoscape v3.9.0 (78). The circular nodes represent unique spacers from bacterial contigs and are colored by taxonomy. The triangular nodes are phage contigs. Solid edges represent the percent nucleotide identity over the entire spacer length. The dashed edge shows the connection between Desulfofustis phage MD01 and *Desulfofustis* sp. PB-SRB1 repeat 01 spacer 64, which is below 80% identity and included in panel C. The nucleotide sequences of phage protospacers within conserved capsid genes that were identified on phage contigs were aligned to (B) *Desulfofustis* sp. PB-SRB1 repeat 01 spacer 103 and (C) *Desulfofustis* sp. PB-SRB1 repeat 01 spacer 64. Spacer 02-11 is the reverse complement of 01-103 and is not shown. The resulting metagenome-wide variation in protospacer sequences from mapping reads to protospacers is shown as sequence logos (73, 79).

To look for evidence of previous phage-bacteria interactions, metagenomic CRISPR spacer sequences were aligned to the eight complete phage genome assemblies (Fig. 2 and 5A). Of the 2,802 unique spacer sequences that were extracted from the merged set of metagenomic reads, 163 unique spacers aligned to seven phage contigs of interest with at least 80% identity over the entire spacer length (14) (Fig. 2 and 5A; Data Set S5). Spacers from three of the four potential host taxa aligned to phage genomes, whereas no spacers from *Flavobacteriales* aligned to any phage genome of interest. *Thiohalocapsa* sp. PB-PSB1 was predicted to be the host of Thiohalocapsa phages MD03 and MD04, *Rhodobacteraceae* sp. A2 was predicted to be the host of Rhodobacteraceae phages MD05, MD06, and MD07, and *Desulfofustis* sp. PB-SRB1 was predicted to be the host of Desulfofustis phages MD01 and MD02 (Fig. 4A; Data Set S5). For two of the most prevalent and abundant phages identified, namely, Desulfofustis phage MD02 (Fig. S3A) and Thiohalocapsa phage MD04 (Fig. S3B), the phage genome and the targeting CRISPR spacer coverages were positively correlated. Moreover, although most host spacers matched to a single virus, two spacers from the *Rhodobacteraceae* host aligned to Thiohalocapsa phage MD04 and Desulfofustis phage MD02 (Fig. 4A). We do not predict these phages to have infected the *Rhodobacteraceae* bacterium, since there was only one alignment each to Thiohalocapsa phage MD04 and Desulfofustis phage MD02, compared to 126 and 3 alignments from the two phage genomes to *Thiohalocapsa* and *Desulfofustis* spacers, respectively (Data Set S5). Additionally, the alignments from the two *Rhodobacteraceae* spacers to Desulfofustis phage MD02 were weaker than the alignments from the *Desulfofustis* spacers (Fig. 4A; Data Set S5). Alignments to multiple host taxa may be due to CRISPR systems targeting motifs that are present in several phage lineages.

The majority of CRISPR spacer-protospacer matches occurred only once in the data set. However, four spacers from *Thiohalocapsa* aligned imperfectly to two distinct protospacers on the genome of Thiohalocapsa phage MD04 (*Thiohalocapsa* sp. PB-PSB1 spacers 09-2, 21-2, 21-11, and 21-164 in Data Set S5). An additional two spacers from *Desulfofustis* sp. PB-SRB1 were reverse complements of each other and targeted the same protospacer sequence on both Desulfofustis phage MD01 and MD02 (*Desulfofustis* sp. PB-SRB1 spacers 01-103 and 02-11) (Fig. 1, 4A, and 4B; Data Set S5). The shared CRISPR targeting of Desulfofustis phage MD01 and MD02 occurred in a conserved gene that was predicted to encode a phage capsid protein (Fig. 2; Data Set S2) (ORFs DPMD01_45 and DPMD02_11, respectively), and corresponded to genomic regions with high read coverage, relative to the remainder of the phage genomes (Fig. 2; Fig. S1). A third spacer (01-64) targeted a distinct protospacer within this same capsid gene on Desulfofustis phage MD02. A third capsid gene from an incomplete viral contig was found to be homologous to these two variants from Desulfofustis phage MD01 and MD02, and it is 92% identical and of similar length. The capsid gene from this incomplete phage contig aligned with the same three spacers targeting Desulfofustis phage MD01 and MD02 (Fig. 5B; Data Set S5). Nucleotide variation at these protospacers, as inferred via read mapping, showed that other variants of these protospacers likely exist in related pink berry phages that are not assembled here (Fig. 5B). We obtained metagenome-wide allelic variants that span the entire capsid gene of Desulfofustis phage MD01, Desulfofustis phage MD02, and the incomplete phage contig 1, and we observed a nearly threefold increase in the number of variants over CRISPR-targeted regions (Fig. S4). Further, the N terminus of this capsid gene, including the protospacer discussed here, is notably absent from the genome of the presumed MD01 ancestor that was identified in a 2011 metagenome (19) (Fig. S2), despite the remainder of the protein being highly conserved. Taken together, these results suggest that this CRISPR-targeted conserved capsid gene is an active site of diversification.

### Horizontal gene transfer between pink berry phages and their hosts.

Desulfofustis phage MD02 and Thiohalocapsa phage MD04 were found to contain discrete regions of high read coverage, compared to the rest of the genome (Fig. 2; Fig. S1). We hypothesized that these regions could be the result of read mapping from homologous regions of bacterial chromosomes or other phage genomes.

The high coverage region on Desulfofustis phage MD02 (genome coordinates 170 to 1760) did not align with any region of the *Desulfofustis* sp. PB-SRB1 host reference genome (GenBank: JAEQMT000000000.1) or with those of any other bacterial genomes in RefSeq. This region also did not successfully align with any other contigs in the coassembly, indicating that the coverage at this region is not the result of the conservation of this sequence among other members of the metagenome. Because of the circularity of these genomes, the high read coverage at these regions could be attributed to terminal redundancy from circular permutation (32, 33).

**FIG 6:**
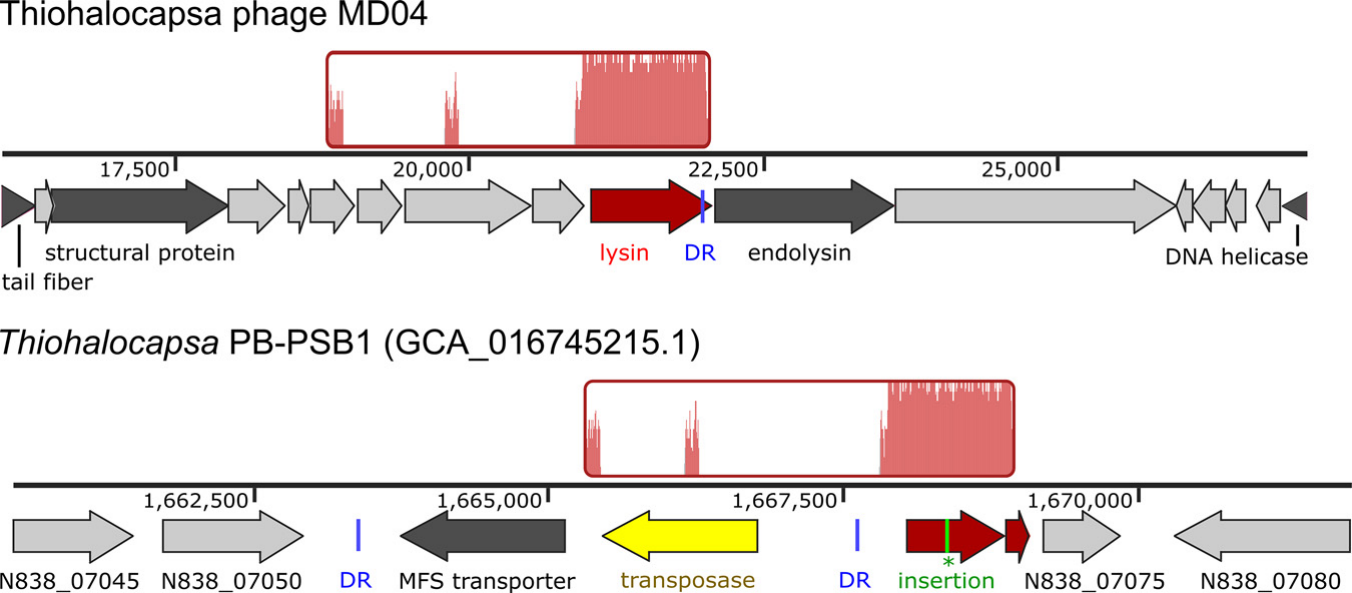
Region of homology between Thiohalocapsa phage MD04 and its host. Locally colinear blocks aligned with Mauve (66) are shown in red, with traces inside representing nucleotide similarity. Genome tracks show genome coordinates and ORFs. Conserved 17 bp direct repeat (DR) sequences are shown in blue. A single-nucleotide insertion (green) in the *Thiohalocapsa* ORF N838_07070 results in a premature stop codon and a pseudogene annotation.

The high coverage region of Thiohalocapsa phage MD04 (genome coordinates 21,035 to 22,077) encodes a glucosaminidase domain-containing protein (ORF TPMD04_36, WBC28587.1) that is predicted to function as the phage lysin. TPMD04_36 is homologous to two predicted ORFs (NCBI N838_07070 and N838_07065) in the *Thiohalocapsa* sp. PB-PSB1 genome (GenBank CP050890.1), and these are adjacent to a predicted transposase (Fig. 6). This observation prompted an investigation into a possible transposon-mediated HGT event between Thiohalocapsa phage MD04 and its host, namely, *Thiohalocapsa* sp. PB-PSB1.

The alignment of the Thiohalocapsa phage MD04 and *Thiohalocapsa* PB-PB1 genomes revealed that the phage lysin gene and the host pseudogene are in frame with each other, except that the host ORF N838_07070 contains a single-nucleotide insertion at position 1,668,396 that results in a premature stop codon (Fig. 6). Using long read sequence data (from [20]), we confirmed that this region of the *Thiohalocapsa* sp. PB-PSB1 genome was correctly assembled and that both the error-corrected long reads and the highly accurate short reads supported this single nucleotide insertion. Upon closer inspection of the region surrounding this pseudogene in the *Thiohalocapsa* sp. PB-PSB1 genome, we observed a transposon of the IS4 family with terminal inverted repeats and numerous direct repeats that were indicative of past transposase activity (Fig. S5; Data Set S6). The Thiohalocapsa phage MD04 genome contains an imperfect copy of a 17-bp direct repeat, differing by only one nucleotide, at the N-terminal of the TPMD04_36 ORF (Data Set S6). Although MD04 was frequently targeted by host CRISPRs (including at protospacers directly adjacent to the lysin gene), no spacer-protospacer alignments were observed within the lysin gene. This is likely the result of selection against CRISPR self-targeting. Taken together, these findings suggest that a past transposon-mediated HGT event may have resulted in the transfer of the phage lysin gene from either Thiohalocapsa phage MD04 or a related ancestral phage to its host.

## DISCUSSION

Marine phages are the most numerous biological components of the global ocean, outnumbering their bacterial hosts by tenfold (3), and they play vital ecosystem roles as predators that turn over organic matter via bacterial lysis (34–37) and as agents of HGT that impact bacterial community structure and function (3, 38–40). Pink berries are marine microbial aggregates with a microscale sulfur cycle, and they have been used as a model system with which to study cryptic biogeochemical cycling (19). Although phages have been identified within the pink berry metagenomes (20), the full diversity of phages and their impacts on pink berry communities remained largely unexplored. Here, we investigated these simple, naturally occurring microbial communities as a model for phage-host coevolution.

We coassembled three pink berry samples, recovering 8 complete phage genomes that spanned a total of 350 Kb and infecting 3 bacterial species within the consortia *Desulfofustis* sp. PB-SBR1, Rhodobacteraceae sp. A2, and *Thiohalocapsa* sp. PSB1. We found that pink berry-associated phages are highly diverse and largely novel, as seven of the eight complete phage genomes that were analyzed failed to cluster with any known phage sequence. One pink berry phage, namely, Desulfofustis phage MD02, is only the second member of the genus *Knuthellervirus.* The other member of the *Knuthellervirus*, namely, Pseudomonas phage PMBT14, infects Pseudomonas fluorescens, which is another marine organism, suggesting that this phage genus infects diverse hosts.

Although the composition of the pink berry bacterial community is similar across individual aggregates, we found that phage presence and abundance were highly heterogeneous across samples. Similar patterns of bacterial homogeneity and concurrent variation in phage populations across samples have been observed in diverse environments, including the human gut (41), the upper ocean (42), and acidic hot springs (43). Pink berries are free-living microbial aggregates that exist at the sediment-water interface of intertidal ponds, with no obvious physical barrier to phage entry into the system. This raises ecological questions about the mechanisms underlying phage ingress into a pink berry aggregate and their persistence within, loss, or exclusion from the community.

CRISPRs are a common phage-resistance system employed by bacteria and archaea (10, 11, 44). We identified 2,731 unique CRISPR spacer sequences from pink berry-associated *Desulfofustis*, *Rhodobacteraceae*, and *Thiohalocapsa* hosts, 163 of which (approximately 6%) target a complete phage genome that we assembled. This discrepancy suggests that the true diversity of phages that are encountered by pink berry-associated bacteria is far greater than what we report here. Seven of the eight investigated phages were targeted by the described pink berry bacterial CRISPR systems, and the eighth, namely, pink berry phage MD08, may infect other pink berry-associated taxa that do not encode CRISPR defenses or for which we do not yet have a high-quality reference genome. Finally, we observed the diversification of a CRISPR-targeted conserved capsid gene, which is inconsistent with diversification across the rest of the phage genomes. Although we cannot establish a causative relationship between CRISPR targeting and phage variation, these observations are consistent with a model of CRISPR-driven evolution causing positive selection in a phage structural protein. We further observed a positive correlation between CRISPR spacer abundance and target phage abundance for two of the most prevalent and abundant phages that were identified. This correlation suggests that these CRISPR spacers are positively selected within individual pink berry consortia, and this result is consistent with observations from diverse microbial systems (45, 46).

Phages and other mobile genetic elements are powerful mediators of HGT (3–5). HGT between bacterial species within the pink berry consortia have been previously reported (20), yet the roles of phages in HGT and bacterial genome evolution in this system remain to be explored. We identified a predicted phage lysin gene that was horizontally transferred from Thiohalocapsa phage MD04 to its *Thiohalocapsa* host, likely via a transposon intermediary. It is unclear whether the lysin gene that is encoded on the *Thiohalocapsa* sp. PB-PSB1 genome is functional; a nonsense mutation in this ORF suggests that it is a pseudogene and was perhaps selected for to avoid the deleterious effects of expressing this potentially lethal protein. Future work should aim to experimentally determine how these phages impact the evolution of individual bacterial hosts and entire pink berry aggregates through HGT.

Taken together, our results demonstrate that pink berry communities contain diverse and variable phage consortia that are highly targeted by host-encoded CRISPR systems. We leveraged metagenomic sequence data to better understand the phage-host coevolution that occurs through CRISPR evasion and HGT. Pink berries offer a simple, yet relatively unexplored, naturally occurring model of phage invasion into and exclusion from microbial communities. The potential roles of phages in pink berry syntrophy and community-wide metabolic exchanges remain to be explored, but it is now clear that phages are notable members of these microbial consortia.

## MATERIALS AND METHODS

### Sampling.

Pink berries, their surrounding sediment, and seawater were collected from pond LS06 (41.57587, −70.63781) in the Little Sippewissett Salt Marsh in Falmouth, MA, on July 17, 2018, using sterile 50 mL conical tubes. Samples LS06-2018-s01 and LS06-2018-s02 each contained one pink berry aggregate, and sample LS06-2018-s03 contained three pink berry aggregates. These samples were transported to the lab and immediately processed for DNA extraction.

### DNA isolation and sequencing.

Pink berry samples were each mechanically homogenized in 1 mL of TE buffer and centrifuged at 1,000 × *g* for 1 min to pellet particulate matter. The supernatant was removed and subjected to a Wizard Genomic DNA Purification Kit (Promega catalog no. A1120), according to the manufacturer’s instructions. Purified DNA was fragmented, and sequencing adapters and barcodes were ligated with a Nextera DNA Flex Library Prep Kit (Illumina catalog no. 20018705) using Nextera DNA CD indexes (Illumina catalog no. 20018708). The DNA yield was measured with a Qubit High Sensitivity dsDNA Assay Kit (ThermoFisher catalog no. Q32851), and DNA from LS06-2018-s01, LS06-2018-s02, and LS06-2018-s03 were pooled at a ratio of 1:1:10. After pooling, the DNA was purified with Ampure XP beads (Beckman Coulter catalog no. A63881), according to the manufacturer’s instructions. DNA sequencing was performed on an Illumina HiSeq 2500 using a 2 × 250nt protocol at the University of Illinois at Urbana-Champaign Roy J. Carver Biotechnology Center.

### Metagenome coassembly and read mapping.

For each sample, the reads were quality checked with FASTQC v0.11.9 (47) and trimmed using Trimmomatic v0.39 (48), with the adapter sequences being removed. Trimmomatic filtered the reads using a sliding window of 4 bp, a minimum average base quality score of 15, a minimum quality score for retention on the leading and trailing ends of 2, and a minimum read length of 100 bases. The resulting trimmed and filtered reads were merged into a single set of reads and coassembled using Metaviral SPAdes v3.15.2 (22) with the default parameters to maximize the recovery of complete, circular phage genomes. To estimate the phage and bacterial abundances, Bowtie2 v2.4.5 (49) was used to map reads from the set of merged reads or from each pink berry sample to assembled phage contigs and to representative host genomes from NCBI BioProject PRJNA684324: *Desulfofustis* sp. PB-SRB1 (GenBank: JAEQMT010000010.1), *Rhodobacteraceae* sp. A2 (GenBank: JAERIM010000001.1), and *Thiohalocapsa* sp. PB-PSB1 (GenBank: CP050890.1) (20). Read mapping statistics were obtained from Bowtie2 alignments using SAMtools v1.15.1 (50).

The phage and bacterial genome abundances were estimated by calculating the reads per kilobase per million mapped reads (RPKM) for each sample. To calculate the RPKM, the read coverage was normalized to the genome length in Kb and to the sequencing depth of the metagenome sample in millions of reads (51). For bacterial MAGs, the RPKM was calculated using the read coverage across all contigs in the MAG and the total length of all contigs in the MAG.

### Phage sequence identification, binning, and annotation.

ViralVerify v1.1 (22) was used with the default settings to categorize the metagenome contigs as putatively bacterial or viral. ViralComplete v1.1 (22) was used with the default settings to identify the viral contigs that represented complete phage genomes. To verify these predictions, VIBRANT v1.2.1 (21) was used with the default settings on the same metagenome contigs. All of the resulting putative viral contigs that were estimated to be complete viral genomes by both prediction tools were targeted for downstream analyses and annotation. vConTACT v2.0 (28) was used with the RefSeq v211 viral database (52) to cluster the viral contigs of interest with existing phage genomes and to approximate phage taxonomy.

The viral contigs of interest were passed through Pharokka v1.0.1 (53), using PHANOTATE v1.5.0 (54) to predict genes and PHROGs v3 (55) to provide initial protein annotations. We also predicted protein functions using Phyre2 (56), BLASTp v2.11.0 (57) with the NCBI RefSeq v211 virus amino acid database and the nonredundant amino acid database (52, 58), and HMMER v3.2.1 (59, 60) with Pfam-a v35.0 (60) and TIGRFAMs v15.0 (61). Phage lifestyle was predicted using BACPHLIP (23). The resulting predictions from each method were manually reviewed for each protein, and a consensus annotation was inferred (Data Set S2). Clinker v0.0.23 (62) was used to identify conserved genes among the phage genomes, which were then aligned against all of the contigs in the metagenome coassembly using tBLASTx v2.11.0 (63). Putative restriction sites were identified using FIMO (64) by searching each phage genome for the predicted recognition sequences of its host’s restriction enzymes (20).

Metagenomic reads from pink berries sampled in 2011 (SRA: SRR13297012) (20) were mapped to the viral contigs of interest with Bowtie2 via the same methods described above. Viral contigs with >90% average coverage by the 2011 reads were aligned against a database of contigs that was previously assembled from the 2011 reads using BLASTn v2.11.0 (57) with the default parameters. Resulting hits to the same subject (i.e., a contig from 2011) that collectively spanned >90% of a query (i.e., a virus from 2018) were considered to be matching viral genomes. Average nucleotide identities (ANI), as calculated by Goris et al. (65), between the viral contigs and their counterpart 2011 contigs were obtained using the ANI calculator (enve-omics.ce.gatech.edu/ani) with the default settings. For instances in which the BLASTn results yielded >1 subject contig per query contig, the resulting subject contigs were binned into a single genome for ANI calculations. Finally, viral genomes with matches to 2011 contigs were then aligned and visualized with Mauve v2015-02-13 (66).

### CRISPR spacer-protospacer analysis and host prediction.

CRISPRclassify v1.1.0 (67) and MinCEd v0.4.2 (68) were used to identify CRISPR repeat sequences from representative genomes of pink berry taxa from NCBI BioProject PRJNA684324: *Desulfofustis* sp. PB-SRB1 (GenBank: JAEQMT010000010.1), Oceanicaulis alexandrii (GenBank: JAERIO000000000.1), *Rhodobacteraceae* sp. A2 (GenBank: JAERIM010000001.1), *Thiohalocapsa* sp. PB-PSB1 (GenBank: CP050890.1) (20, 69), and *Flavobacteriales* bacterium (GenBank: DNTB01000031.1). The identified repeats from each tool were combined and dereplicated to obtain a list of repeats found in the genomes of pink berry taxa. Since CRISPR arrays are often misassembled with short read data, putative spacer sequences from the trimmed and filtered reads of each LS06-2018 metagenome were identified using NARBL (14) with the dereplicated set of repeats that was identified from the reference genomes, an approximate repeat size of 36, and a minimum coverage of supporting neighbor spacers of 2. Since repeat sequences are highly conserved between bacterial species (70), any spacer identified by NARBL was inferred to belong to the same species as the reference genome from which its associated repeat came. The resulting spacers were aligned to the viral contigs of interest using Spacerblast v0.7.7 (71). Viral contigs that aligned to spacers with at least 80% identity over the full length of the spacer were considered to have a host match with the bacterium whose genome contained the spacer. The merged set of filtered and trimmed reads were aligned to spacers identified by NARBL with Bowtie2, and read coverage statistics were obtained by using SAMtools as described above.

Metagenome reads were mapped to the regions containing conserved phage genes that were identified by using Clinker or tBLASTx above, with the spacer-protospacer alignments being found by using Bowtie2. Mapped reads were converted to multiple sequence alignments using SAM4WebLogo in JVarkit v2021.10.13 (72), and sequence logos were visualized using WebLogo (73). Allele variants for conserved phage genes were called by using Snippy v3.2 (github.com/tseemann/snippy), with the default settings, on metagenome reads. Variants that resulted before the “–mincov” and “–minfrac” filters were applied were used downstream to maximize the number of possible variants recovered. Variant statistics were obtained and visualized using vcfR v1.13.0 (74) with the default settings, except that 100 bp size windows were used instead of 1,000 bp size windows.

### Horizontal gene transfer analysis.

Uneven sequence coverage patterns on phage genomes are sometimes attributed to HGT between the phage and its host genome, especially if the region aligns with the host genome and/or contains genes that facilitate HGT (75). Viral genomes of interest and their coverages from the merged set of metagenome reads were visualized in IGV v2.11.4 (76). Any discrete regions of the viral genomes of interest that had much higher read coverage (>3×) than the surrounding region were aligned to their predicted host genomes, if available, using BLASTn v2.11.0 (63). Predicted regions of HGT between phages and their hosts were aligned with Mauve v2015-02-13 (66). Conserved repeats flanking a putative transposon were initially identified via reciprocal BLASTn using “blastn-short”. The transposon region with its inverted repeats in the *Thiohalocapsa* sp. PB-PSB1 genome was identified with ISEScan v1.7.2.3 (77), and other repeats were identified and annotated manually in Geneious Prime v2022.1.1 (www.geneious.com/prime).

### Data availability.

The DNA sequencing reads from this study were deposited into the NCBI SRA under PRJNA907316. The assembled phage genomes were deposited into the NCBI GenBank under the following accession numbers: OP947158.1 (Desulfofustis phage MD01), OP947159.1 (Desulfofustis phage MD02), OP947165.1 (Thiohalocapsa phage MD03), OP947166.1 (Thiohalocapsa phage MD04), OP947161.1 (Rhodobacteraceae phage MD05), OP947162.1 (Rhodobacteraceae phage MD06), OP947163.1 (Rhodobacteraceae phage MD07), OP947164.1 (pink berry phage MD08), and OP947160.1 (pink berry virus MD00).
